# Proteomics study on the effect of silybin on cardiomyopathy in obese mice

**DOI:** 10.1038/s41598-021-86717-x

**Published:** 2021-03-30

**Authors:** Fei Wang, Zelin Li, Tiantian Song, Yujiao Jia, Licui Qi, Luping Ren, Shuchun Chen

**Affiliations:** 1 Department of Endocrinology, Hebei General Hospital, Graduate School of Hebei Medical University, Shijiazhaung, China; 2 Department of Endocrinology, Hebei General Hospital, Graduate School of Hebei North University, Shijiazhaung, China; 3 Department of Endocrinology, Hebei General Hospital, Shijiazhaung, 050000 Hebei China; 4 Department of Endocrinology, Hebei General Hospital, Graduate School of Hebei North University, No. 348 Heping West Road, Shijiazhaung, 050000 Hebei China

**Keywords:** Computational biology and bioinformatics, Cardiology

## Abstract

Due to the increase in the number of obese individuals, the incidence of obesity-related complications such as cardiovascular disease and type 2 diabetes is higher. The aim of the present study was to explore the effects of silybin on protein expression in obese mice. Firstly, serum was collected, and it was used to detect serum lipids and other serological indicators. Secondly, total protein from epididymal adipose tissue was extracted for differential expression analysis by quantitative tandem mass tag (TMT) combined with liquid chromatography-tandem mass spectrometry (LC–MS/MS), followed by bioinformatics and protein–protein interaction (PPI) network analyses of these proteins. Lastly, real-time polymerase chain reaction (RT-PCR) and parallel reaction monitoring (PRM) were used to further validate the expression of identified differentially expressed proteins (DEPs) at the mRNA and protein level, respectively. The results revealed that silybin could improve abnormal lipid metabolism caused by the high fat diet in obese mice. A total of 341, 538 and 243 DEPs were found in the high fat/control (WF/WC), silybin/high fat (WS/WF) and WS/WC groups, respectively. These DEPs mainly participated in lipid metabolism and energy metabolism. Notably, tropomyosin 1 (TPM1), myosin light chain 2 (MYL2), myosin heavy chain 11 (MYH11) and other DEPs were involved in hypertrophic cardiomyopathy, dilated cardiomyopathy and other pathways. Silybin could protect cardiac function by inducing the protein expression of TPM1, MYL2 and MYH11 in the adipose tissue of obese mice.

## Introduction

In the last several years, the number of patients with obesity and who are overweight has rapidly increased. According to the World Health Organization, 47% of the population in Ireland are expected to be obese by 2030^1^. Numerous studies indicate that obesity will increase the morbidity and mortality of cardiovascular disease, and this link will intensify in cases where obesity coexists with other cardiovascular risk factors^2^. Certain studies have proposed the concept of obese cardiomyopathy, that is, the change in the myocardium that is associated with obesity, but is not associated with coronary artery disease, hypertension or other comorbidities^3,4^. The pathogenesis of cardiomyopathy caused by obesity is complex and not fully understood. The known mechanisms include cardiac hemodynamic changes, myocardial lipotoxicity, metabolic disorders, neurohumoral disorders and small blood vessel diseases^5,6^, which can lead to abnormalities in the structure and function of the heart, and eventually lead to heart failure. The metabolic changes observed in the heart of patients with obesity-induced cardiomyopathy appear at the early stages, and usually precede functional changes such as ventricular dysfunction^7^.

There is a close association between visceral adipose tissue and heart metabolism. Adipose tissue can not only store energy, but also secrete several types of adipokine that regulate adipocyte differentiation, oxidative stress, inflammation and apoptosis^8^. Once the adipose tissue is stored beyond its threshold, it will deposit ectopically in important organs such as the liver, pancreas and heart, which ultimately affects organ function. Lipid overload in cardiomyocytes can cause myocardial lipotoxicity and eventually lead to cardiac dysfunction^5^. In obese patients, the epicardial adipose tissue may promote myocardial fibrosis. Previous studies have shown that reducing free fatty acids (FFAs) levels in adipose tissue can rapidly reduce cardiac lipid storage and improve cardiac contractility in patients with type 2 diabetes^9^. In addition to regulating insulin sensitivity, adiponectin can also inhibit cardiac remodeling. Leptin, which is positively correlated with body mass index (BMI) and degree of obesity, plays a cardioprotective role by regulating lipotoxicity^10^. Forkhead box O3 (Foxo3a) is a key transcription factor against hypertrophic growth. In obese mice, high fat diet can cause myocardial hypertrophy by down-regulating Foxo3a^11^.

The majority of studies on cardiomyopathy caused by obesity focus on myocardial lipotoxicity, metabolic disorders and energy metabolism. Notably, myocardial cytoskeleton proteins are important for maintaining the structural and functional integrity of the myocardium^12^. However, there are few studies on cytoskeletal proteins associated with cardiomyopathy caused by obesity. It has been demonstrated that mutations in genes encoding cytoskeletal proteins, including tropomyosin 1 (TPM1) and myosin light chain 2 (MYL2), can cause dilated cardiomyopathy with impaired contractility and diastolic function^13,14^. Proteomics analysis of heart tissue of broilers with sudden death syndrome revealed that TPM1, myosin heavy chain 1 (MYH1) and other myocardial contractile proteins were differentially expressed^15^. Previous studies have found that the level of cardiac lipids is negatively correlated with the gene expression of myocardial contraction-related protein, which indicates that myocardial steatosis may be involved in the occurrence and development of obese cardiomyopathy by affecting the function of myocardial contraction^16^. However, there are few studies on the changes in cytoskeletal proteins associated with cardiomyopathy caused by obesity.

Silybin, one of the polyphenolic antioxidants in the *Compositae* family, is widely used in the clinic as a classic hepatoprotective agent. However, the mechanism by which silybin protects the heart has been rarely studied. Previous studies have shown that silybin has a protective effect on hypertension, atherosclerosis and cardiotoxicity caused by oxidative stress^17^. Silybin can inhibit the activation of NF-κB and reduce the production of epidermal growth factor receptor (EGFR) to improve myocardial hypertrophy^18^. Silybin can also protect cardiomyocytes from cardiotoxicity caused by chemicals by reducing lipid peroxidation, increasing antioxidant enzyme levels, preventing apoptosis^19^, and restoring atherosclerosis by reducing triglycerides and low-density lipoprotein (LDL) levels while increasing high-density lipoprotein (HDL) levels^20^. To date, the majority of studies have focused on the role of silybin in cardiac function by improving myocardial lipid metabolism and energy metabolism. However, few studies have reported the role of silybin on contractile proteins involved in the heart such as TPM1 and MYL2.

In our previous study, it was found that differentially expressed proteins (DEPs) in obese mice were enriched not only in lipid metabolism and energy metabolism pathways, but also in hypertrophic cardiomyopathy and dilated cardiomyopathy pathways. In addition, experimental data showed that proteins involved in myocardial contraction were significantly elevated after silybin intervention^21^. However, few studies have reported the role of silybin in metabolism-related contractile proteins in cardiomyopathy. Therefore, in order to explore the changes in heart contractile proteins and to study the effect of silybin on cardiac contractile protein, the present study intended to use the method of isotope labeling tandem mass tag (TMT) combined with liquid chromatography-tandem mass spectrometry (LC–MS/MS) to explore the expression of proteins involved in cardiovascular disease in obese mice and the influence of silybin on the expression of these proteins.

## Results

### Silybin improves blood lipids in obese mice

**Figure 1:**
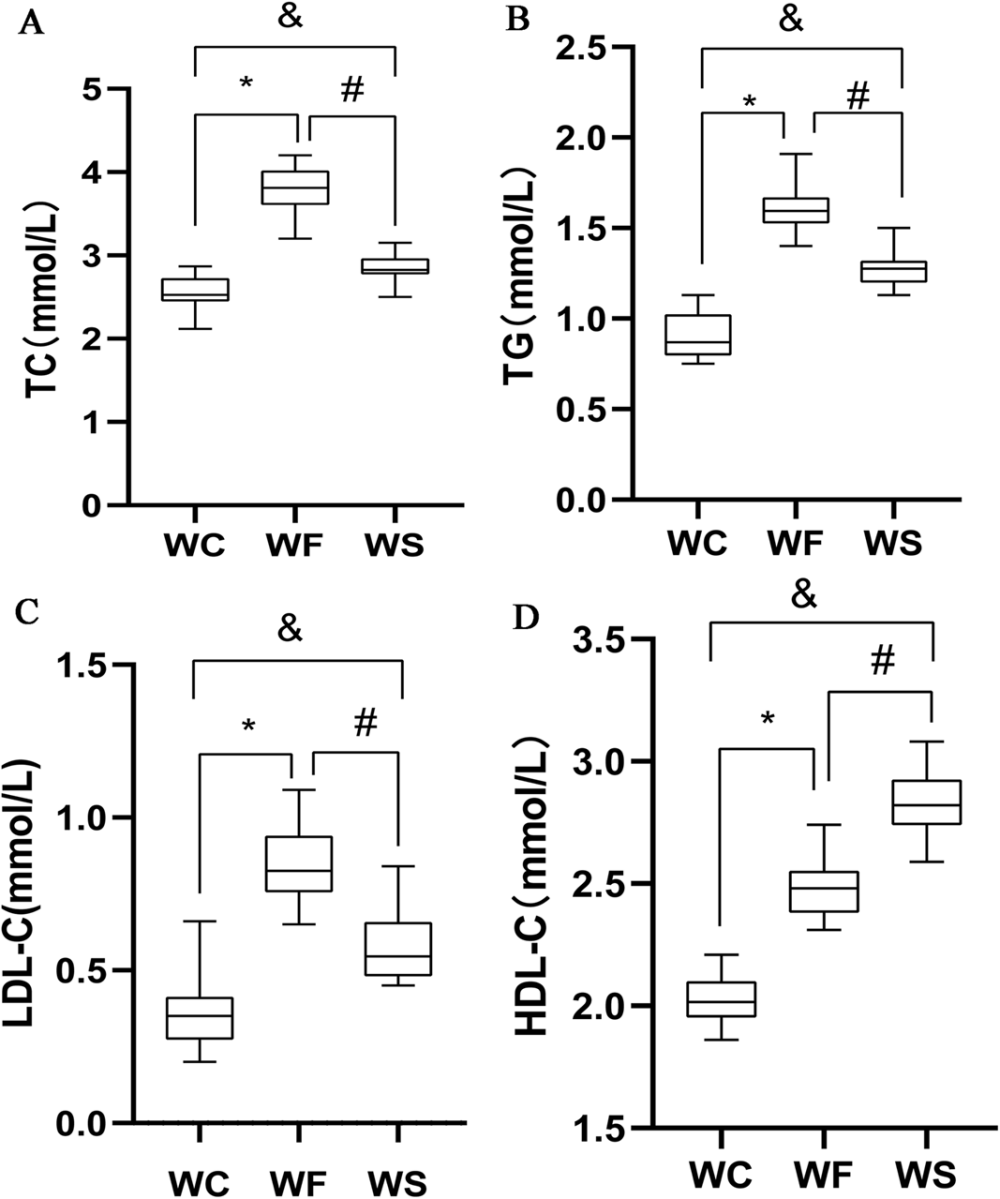
Concentrations of **(A)** total cholesterol, **(B)** triglycerides, **(C)** low-density lipoprotein-cholesterol and **(D)** high-density lipoprotein-cholesterol in the WC, WF and WS groups. *P < 0.05 WF vs. WC, ^#^P < 0.05 WS vs. WF, ^&^P < 0.05 WS vs. WC.

The concentrations of total cholesterol (TC), triglycerides (TG), low density lipoprotein cholesterol (LDL-C), and high density lipoprotein cholesterol (HDL-C) were determined in the serum of mice in the control group (normal diet, WC), high-fat group (high-fat diet, WF) and silybin group (silybin + high-fat diet, WS). The fat metabolism of mice fed with high-fat diet was abnormal. Compared with those of the WC group, the expression levels of adipose ingredients in the WF group were notably increased (P < 0.05). After silybin intervention, the expression levels of TC, TG and LDL-C in the WS group were notably decreased (P < 0.05) compared with those of the WF group, while HDL-C was notably increased (Fig. 1).

### Qualitative and quantitative analyses of protein in adipose tissue

**Figure 2:**
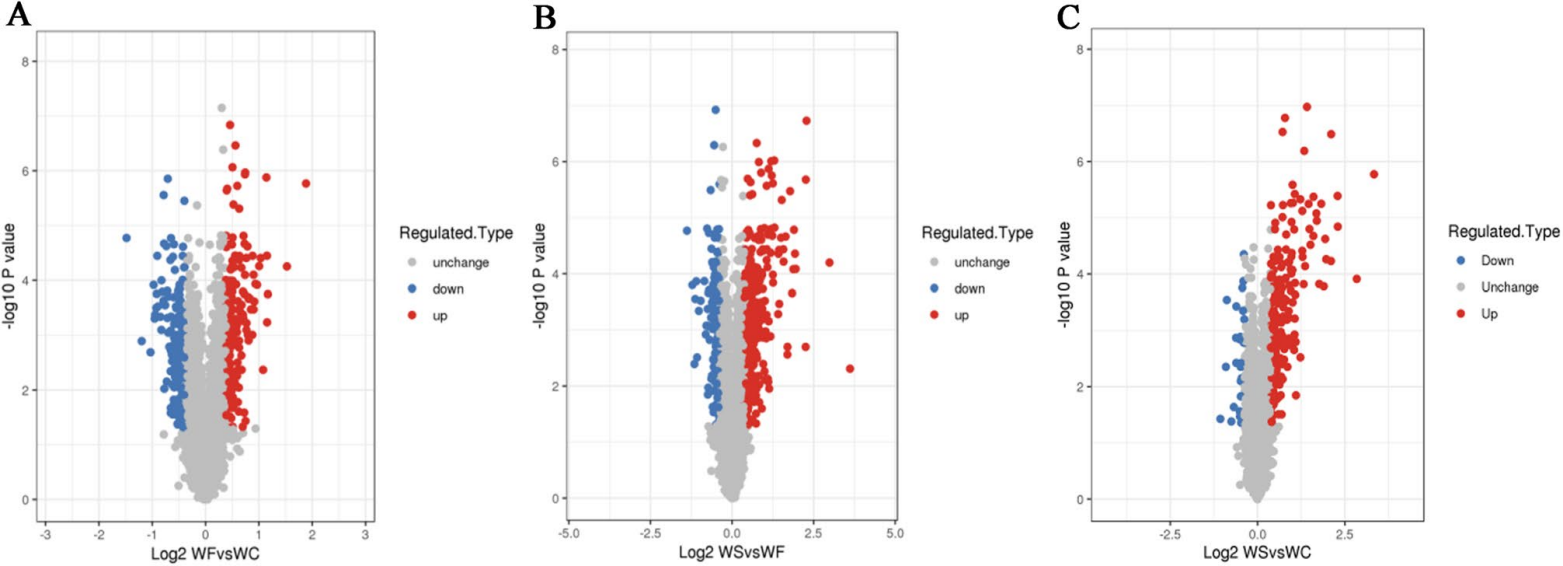
Volcano plots of differentially expressed proteins in the **(A)** WF/WC, **(B)** WS/WF and **(C)** WS/WC groups. The horizontal axis is the relative quantitative value of protein after log2 conversion, and the vertical axis is the value of the P-value after − log10 conversion. Red and blue dots indicates significantly up-regulated and down-regulated proteins, respectively.

**Figure 3:**
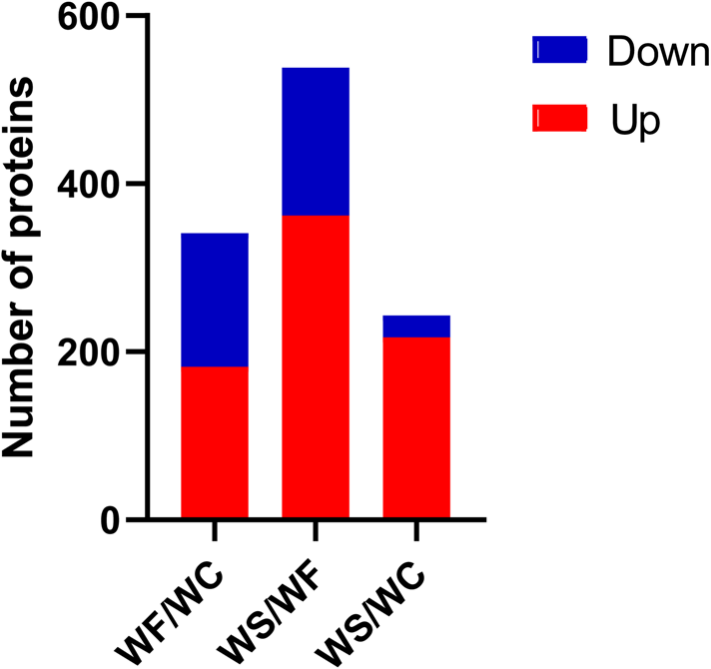
Histogram of the distribution of differentially expressed proteins in the WF/WC, WS/WF and WS/WC groups. Red and blue indicate significantly up-regulated and down-regulated proteins, respectively.

Mass spectrometry (MS) analysis revealed in total 30,0152 spectrum proteins, 45,830 matched-spectrum proteins, 30,121 peptides, 29,108 unique peptides, 5108 identified proteins and 4623 quantifiable proteins. According to the literature^22^, the absolute value of differential multiple > 1.0 was generally selected for differential proteins. In addition, in our previously published article, the threshold was also set to 1.3^21^. The aim of the present study was to investigate the expression of proteins involved in obese cardiomyopathy in obese mice and the effect of silybin on these proteins. The mechanism of action of silybin on obese cardiomyopathy was further investigated. Combined with the present experimental data, it was found that the majority of the key proteins involved in obesity cardiomyopathy, myocardial lipid metabolism and energy metabolism, as well as other key proteins, had an absolute value of the change multiple of > 1.3. After data analysis, the change threshold of DEPs was determined. An increase in expression greater than 1.3-fold was considered up-regulation, while a decrease in expression of great than 1.3-fold was considered down-regulation^21^. The volcano plot of DEPs is shown in Fig. 2. There were 341 DEPs in the WF/WC group, containing 182 up-regulated proteins and 159 down-regulated proteins. There were 538 DEPs in the WS/WF group, containing 362 up-regulated proteins and 176 down-regulated proteins. There were 243 DEPs in the WS/WC group, containing 217 up-regulated proteins and 26 down-regulated proteins (Fig. 3).

### Functional enrichment analysis of DEPs

**Figure 4:**
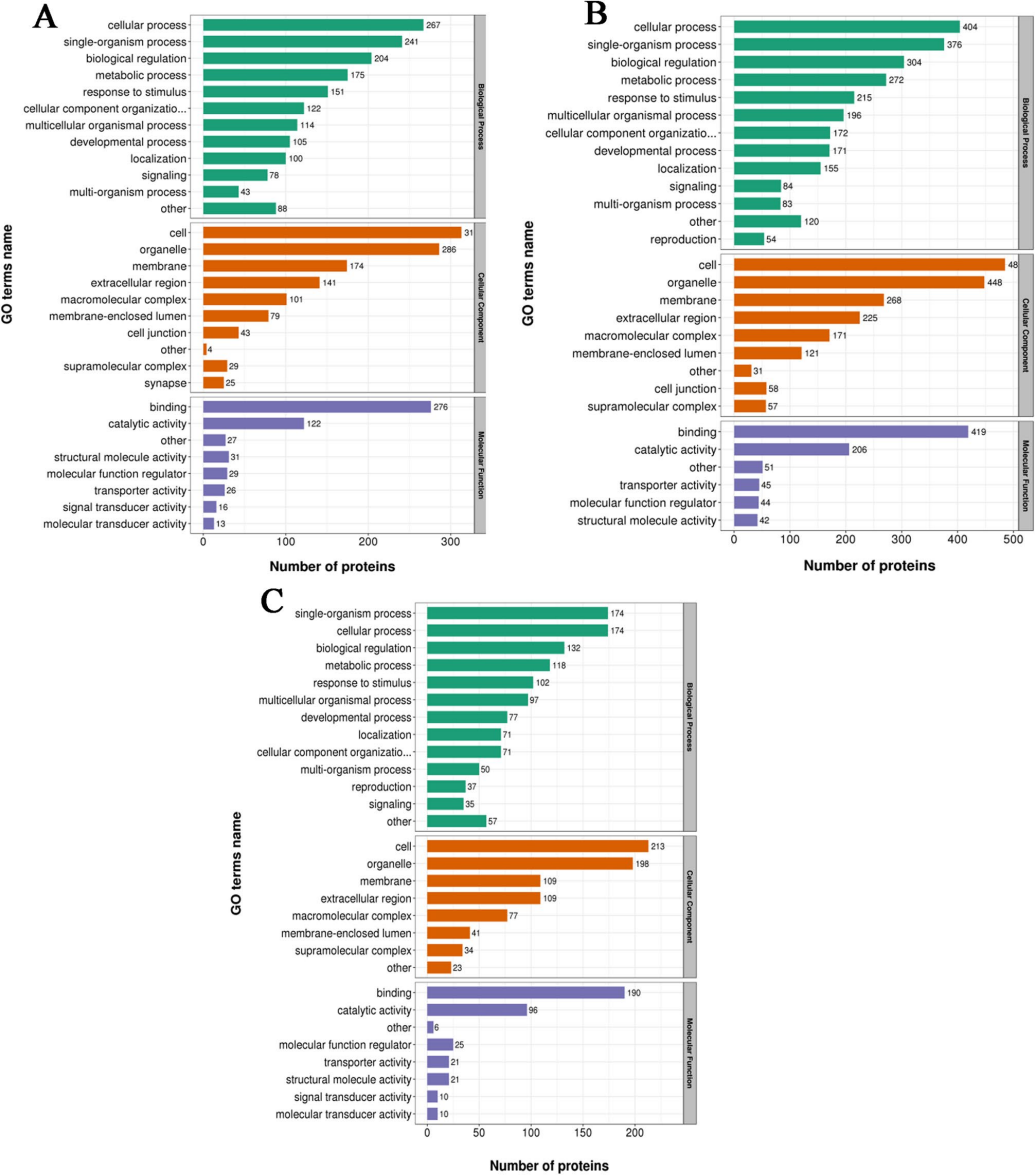
Gene Ontology functional analysis of differentially expressed proteins in the **(A)** WF/WC, **(B)** WS/WF and **(C) **WS/WC groups. Green, red and purple represent biological process, cellular component and molecular function, respectively.

A total of 4623 identified DEPs were imported into Interport Scan software for gene ontology (GO) analysis. In terms of biological process (BP), DEPs in the WF/WC group were involved in cellular processes (267 DEPs), single-cell processes (241 DEPs) and biological regulation (204 DEPs). In terms of cellular component (CC), DEPs in the WF/WC group were distributed in the cytoplasm (313 DEPs), organelles (286 DEPs) and cell membranes (174 DEPs). In terms of molecular function (MF), 276 DEPs were associated with integrated functions and 122 DEPs were associated with catalytic functions (Fig. 4A). In terms of BP, DEPs in the WS/WF group were involved in cellular processes (404 DEPs), single-cell processes (376 DEPs) and biological regulation (304 DEPs). In terms of CC, DEPs in the WS/WF group were distributed in the cytoplasm (485 DEPs), organelles (448 DEPs) and cell membranes (268 DEPs). In terms of MF, 419 DEPs were associated with integrated functions and 206 DEPs were associated with catalytic functions (Fig. 4B). In terms of BP, DEPs in the WS/WC group were involved in cellular processes (174 DEPs), single-cell processes (174 DEPs) and biological regulation (132 DEPs). In terms of CC, DEPs in the WS/WC group were distributed in the cytoplasm (213 DEPs), organelles (196 DEPs) and cell membranes (109 DEPs). In terms of MF, 190 DEPs were associated with integrated functions and 96 DEPs were associated with catalytic functions (Fig. 4C). Enrichment analysis revealed that DEPs are mainly involved in lipid metabolism and energy metabolism.

**Figure 5:**
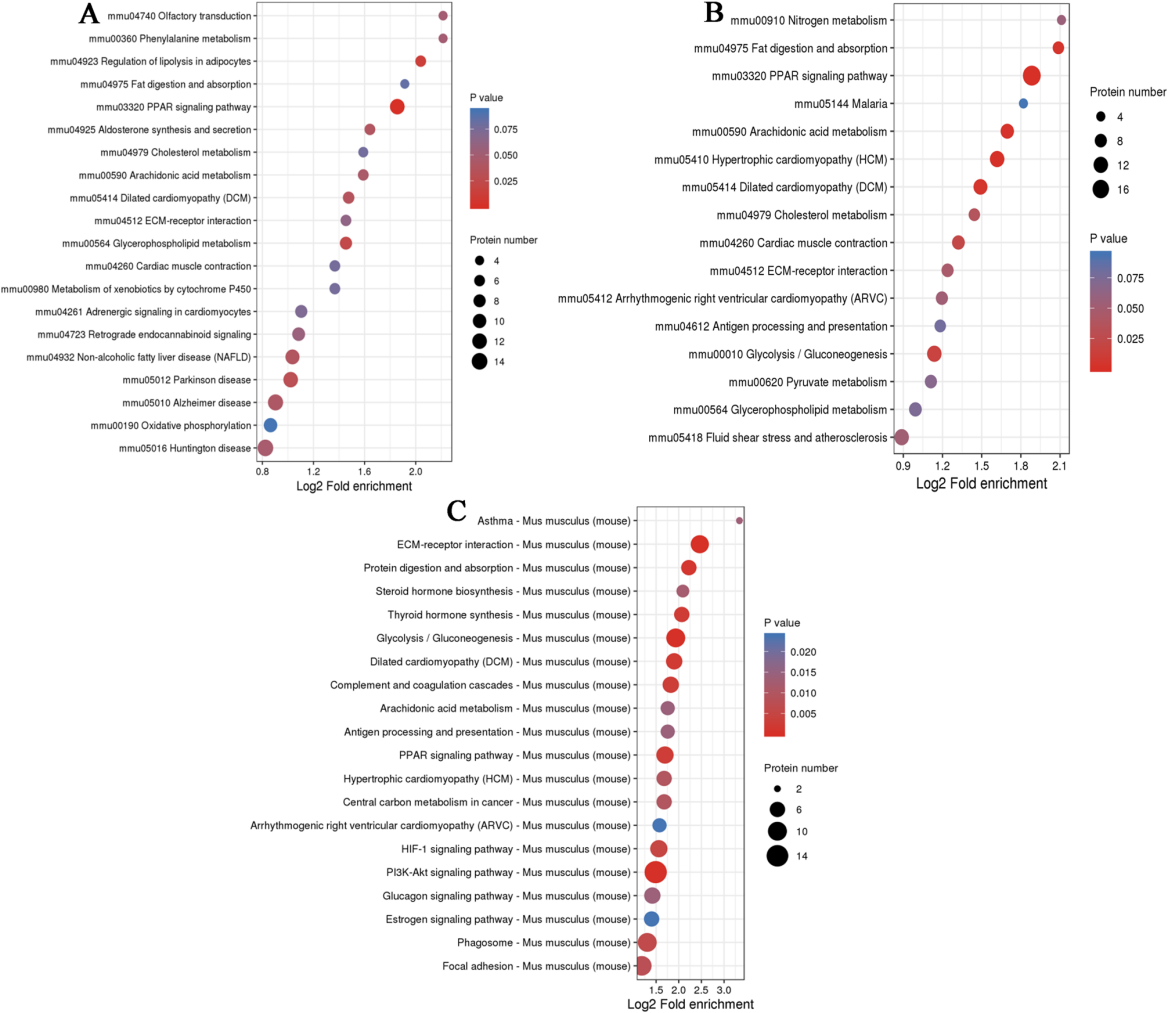
Enriched bubble charts of Kyoto Encyclopedia of Genes and Genomes pathway analysis of differentially expressed proteins in the **(A)** WF/WC, **(B)** WS/WF and **(C)** WS/WC groups.

**Figure 6:**
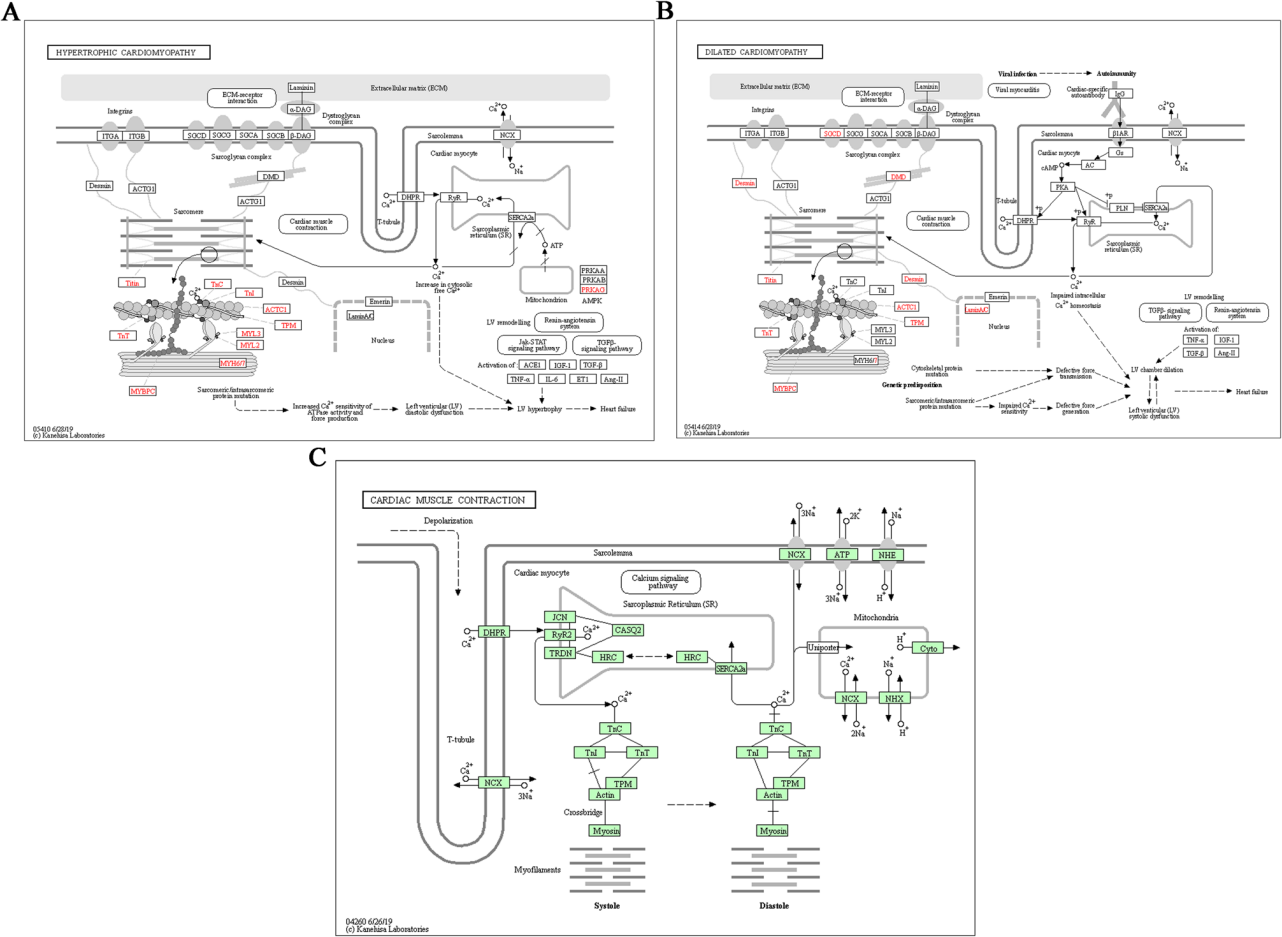
Signaling pathways of hypertrophic cardiomyopathy, dilated cardiomyopathy and cardiac muscle contraction. Diagrams created by Kanehisa Laboratories and used with permission.

In addition, the DEPs were used for Kyoto Encyclopedia of Genes and Genomes (KEGG) analysis through the KEGG Mapper software^23–25^. It was found that the DEPs of the WF/WC and WS/WF groups were significantly enriched in the peroxisome proliferator activated receptor (PPAR) pathway (Fig. 5A,B), while the DEPs in the WS/WC group were mainly concentrated in the PI3K-Akt signaling, glycolysis/gluconeogenesis and PPAR pathways (Fig. 5C). It was also found that the DEPs of the three comparative groups were involved in hypertrophic cardiomyopathy and dilated cardiomyopathy pathways. Notably, it was found that MYH11, TPM1 and MYL2 were involved in cardiac contraction according to KEGG analysis (Table 1). The hypertrophic cardiomyopathy pathway, dilated cardiomyopathy pathway and cardiac muscle contraction maps are shown in Fig. 6.

**Table 1:** MYL2, TPM1 and MYH11 are involved in the signaling pathway.

KEGG pathway	Fisher's exact test P value	Regulated Type	P value	Gene name	Group name
Focal adhesion	0.376447144	Down	0.0126582	Myl2	WF/WC
Cardiac muscle contraction	0.004337664	Down	0.0126582	Myl2	WF/WC
Dilated cardiomyopathy (DCM)	0.340131952	Down	0.0126582	Myl2	WF/WC
Hypertrophic cardiomyopathy (HCM)	0.340131952	Down	0.0126582	Myl2	WF/WC
Regulation of actin cytoskeleton	0.249168461	Down	0.0126582	Myl2	WF/WC
Adrenergic signaling in cardiomyocytes	0.535277442	Down	0.0126582	Myl2	WF/WC
Cardiac muscle contraction	0.001323273	Up	4.417E-05	Myl2	WS/WF
Dilated cardiomyopathy (DCM)	0.009387349	Up	4.417E-05	Myl2	WS/WF
Hypertrophic cardiomyopathy (HCM)	0.002327684	Up	4.417E-05	Myl2	WS/WF
Regulation of actin cytoskeleton	0.080057341	Up	4.417E-05	Myl2	WS/WF
Adrenergic signaling in cardiomyocytes	0.065357402	Up	4.417E-05	Myl2	WS/WF
Regulation of actin cytoskeleton	0.079310435	Up	0.0003173	Myl2	WS/WC
Adrenergic signaling in cardiomyocytes	0.20937049	Up	0.0003173	Myl2	WS/WC
Apelin signaling pathway	0.71190969	Up	0.0003173	Myl2	WS/WC
Dilated cardiomyopathy (DCM)	0.004523248	Up	0.0003173	Myl2	WS/WC
Tight junction	0.164766541	Up	0.0003173	Myl2	WS/WC
Hypertrophic cardiomyopathy (HCM)	0.004523248	Up	0.0003173	Myl2	WS/WC
Focal adhesion	0.020708408	Up	0.0003173	Myl2	WS/WC
Leukocyte transendothelial migration	0.446608147	Up	0.0003173	Myl2	WS/WC
Cardiac muscle contraction	0.057573801	Up	0.0003173	Myl2	WS/WC
Cardiac muscle contraction	0.004337664	Down	0.0004224	Tpm1	WF/WC
Dilated cardiomyopathy (DCM)	0.340131952	Down	0.0004224	Tpm1	WF/WC
Hypertrophic cardiomyopathy (HCM)	0.340131952	Down	0.0004224	Tpm1	WF/WC
Adrenergic signaling in cardiomyocytes	0.535277442	Down	0.0004224	Tpm1	WF/WC
Cardiac muscle contraction	0.001323273	Up	0.0001442	Tpm1	WS/WF
Dilated cardiomyopathy (DCM)	0.009387349	Up	0.0001442	Tpm1	WS/WF
Hypertrophic cardiomyopathy (HCM)	0.002327684	Up	0.0001442	Tpm1	WS/WF
Adrenergic signaling in cardiomyocytes	0.065357402	Up	0.0001442	Tpm1	WS/WF
Vascular smooth muscle contraction	0.234643781	Down	0.0001564	Myh11	WF/WC
Regulation of actin cytoskeleton	0.249168461	Down	0.0001564	Myh11	WF/WC
Vascular smooth muscle contraction	0.050993771	Up	0.0031951	Myh11	WS/WF
Regulation of actin cytoskeleton	0.080057341	Up	0.0031951	Myh11	WS/WF

*KEGG* Kyoto encyclopedia of genes and genomes.

### Protein–protein interaction network analysis

**Figure 7:**
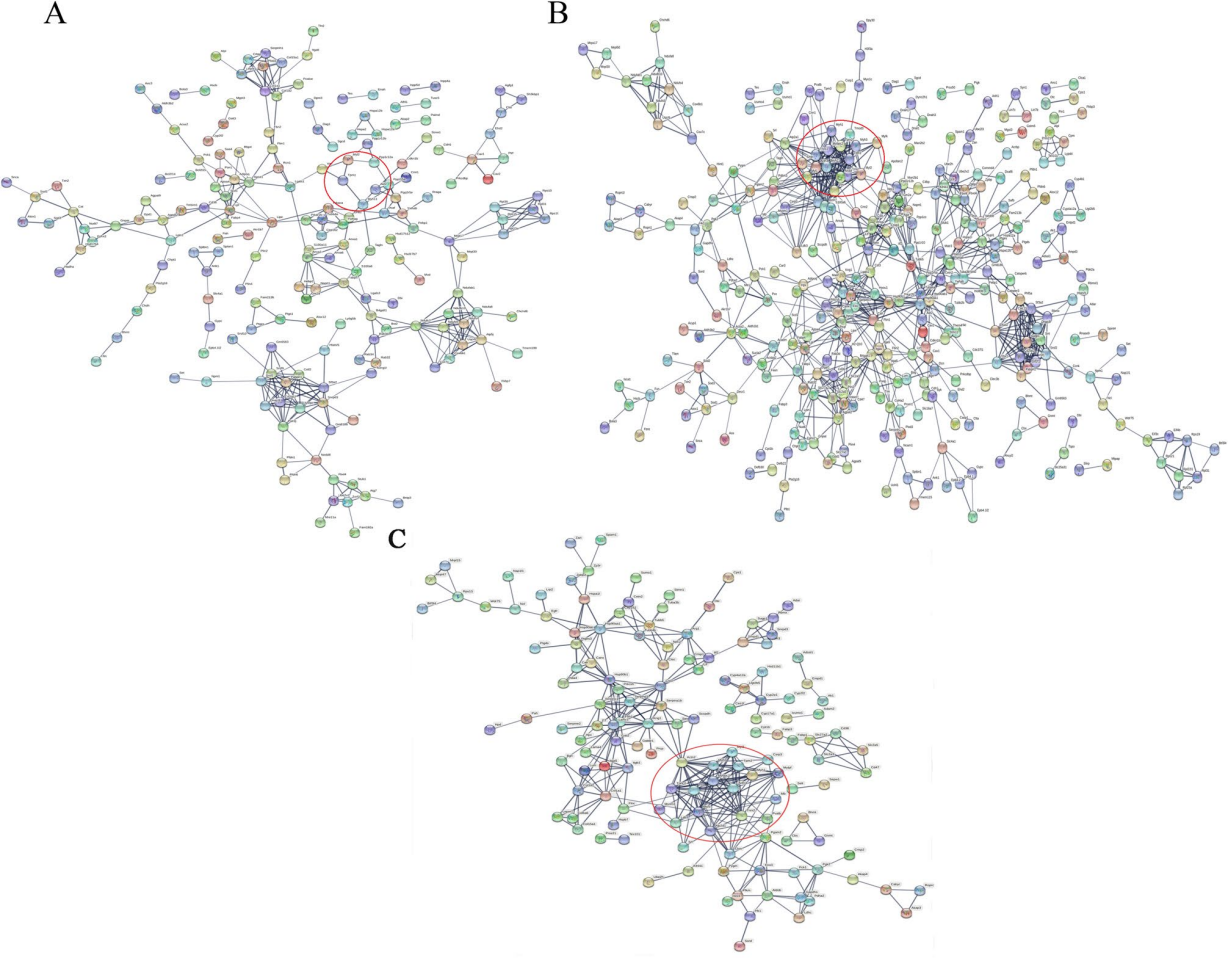
Protein–protein interaction network of differentially expressed proteins in the **(A)** WF/WC, **(B)** WS/WF and **(C)** WS/WC groups. Red circle represents the interaction network between tropomyosin 1 (TPM1), myosin light chain 2 (MYL2) and myosin heavy chain 11 (MYH11).

The protein–protein interaction (PPI) network of DEPs showed that TPM1, MYL2 and MYH11 were highly correlated in the WF/WC and WS/WF groups (Fig. 7). Although only MYL2 was found in the PPI of the WS/WC group, MYL was strongly associated with the family genes of MYH and TPM (Fig. 7C).

### In vitro validation of the mRNA expression of MYH11, TPM1, and MYL2

**Figure. 8:**
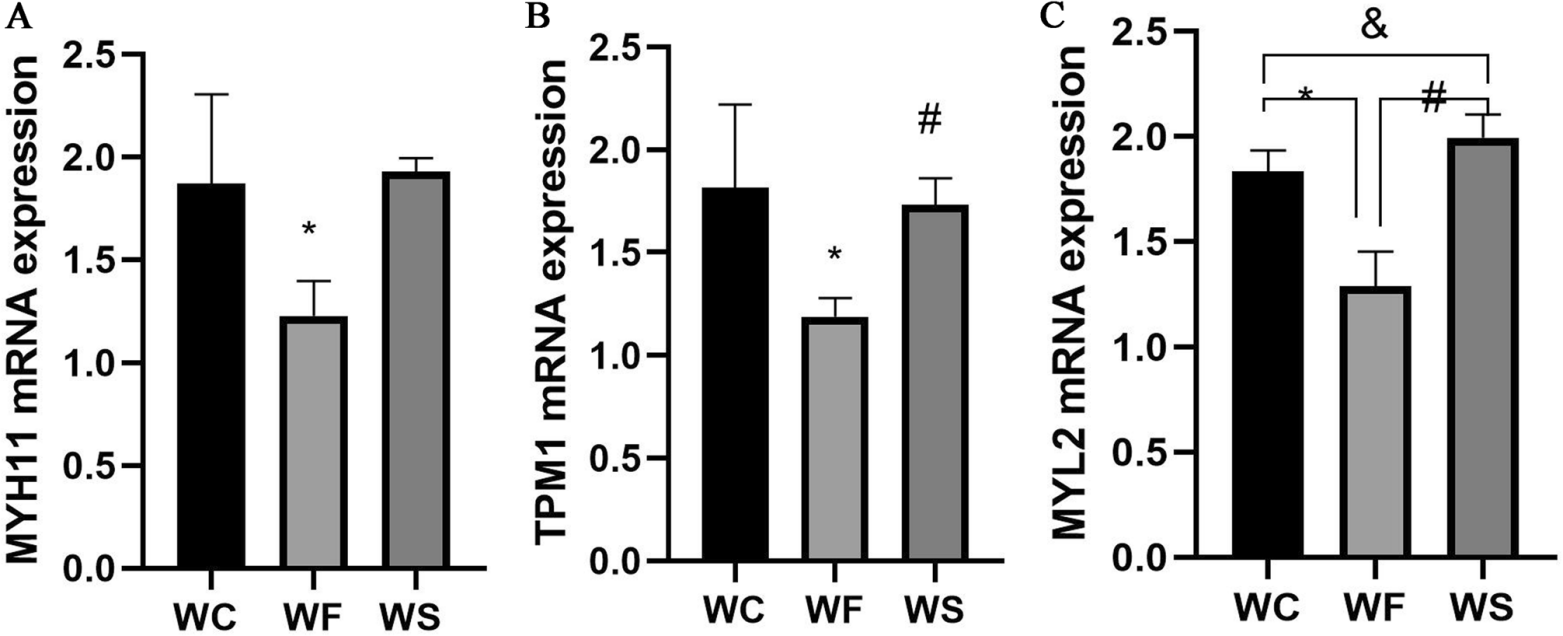
Reverse transcription-quantitative PCR validation of myosin heavy chain 11 (MYH11), tropomyosin 1 (TPM1) and myosin light chain 2 (MYL2) in adipose tissue samples of WC, WF and WS groups. **(A)** The mRNA expression of MYH11 in the group of WC, WF and WS; **(B)** the mRNA expression of TPM1 in the group of WC, WF and WS; **(C)** the mRNA expression of MYL2 in the group of WC, WF and WS. *P < 0.05 WF vs. WC, ^#^P < 0.05 WS vs. WF, ^&^P < 0.05 WS vs. WC.

KEGG analysis revealed that TPM1 and MYL2 were involved in the hypertrophic cardiomyopathy (HCM) pathway. Therefore, they were selected for reverse transcription-quantitative PCR (RT-qPCR) verification. In addition, according to previous studies, MYH11 plays a regulatory role in cardiac function^26,27^. Furthermore, MYH11 was highly correlated with TPM1 and MYL2 in the PPI network of WS/WF and WF/WC. Thus, MYH11 was also selected for RT-qPCR analysis. The primers used are shown in Table 2. The results showed that the expression of MYH11, TPM1 and MYL2 was down-regulated in the WF group compared with that of the WC group. Notably, the addition of silybin (WS group) up-regulated the mRNA expression of MYH11, TPM1 and MYL2 compared with that of the WF group. In addition, the expression levels of MYH11 and TPM1 in the WS group were basically restored to the same level as those in the WC group after silybin treatment, and MYL2 expression was higher than that in the WC group (Fig. 8).

**Table 2:** Sequences of the primers used in reverse transcription-quantitative PCR.

Primer name	Primer sequence (5′ to 3′)
β-ACTIN-F(internal reference)	5-GGCTGTATTCCCCTCCATCG-3
β-ACTIN-R(internal reference)	5-CCAGTTGGTAACAATGCCATGT-3
TPM1-F	5-GGCCCAGGAGCCGTGACTTCC-3
TPM1-R	5-TTCTGACCACTTTTTAAACGGTTTATTTCT-3
MYL2-F	5-AGCCAGCCAGGCAGCCTGGG-3
MYL2-R	5-GCCAGAGCCAAGACTTCCTG-3
MYH11-F	5-AGCTAGAGTGTAATGGAGAGTGTG-3
MYH11-R	5-CTTTCTCGTGACTTACAACCTTTTAT-3

### Verification of MYH11, TPM1 and MYL2 expression at the mRNA level

**Figure 9:**
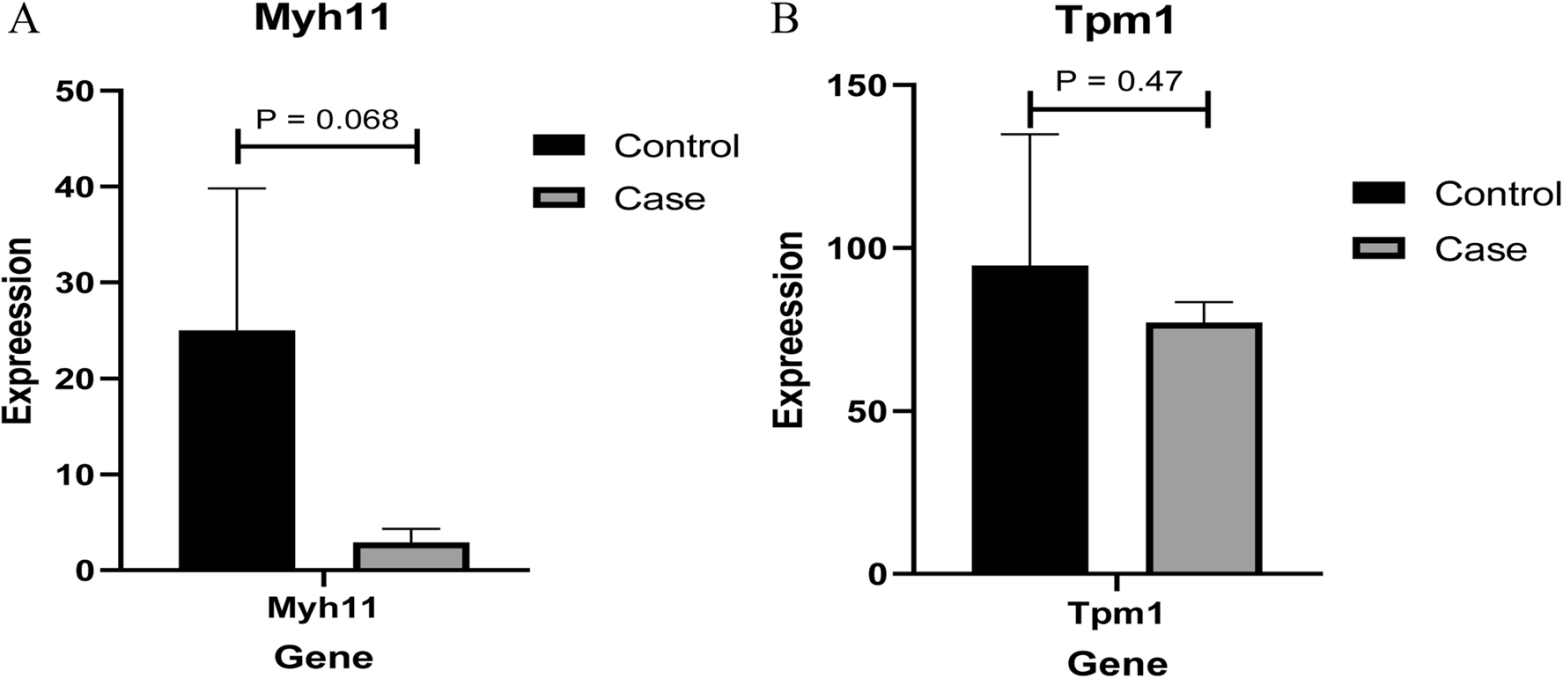
Electronic verification of differentially expressed proteins at the mRNA level. **(A)** The mRNA expression result of myosin heavy chain 11 (MYH11); **(B)** The mRNA expression result of tropomyosin 1 (TPM1). Control and case groups represent the standard group and the high-fat and high-energy feed groups, respectively. P < 0.05 was considered to indicate a statistically significant different.

The mRNA expression levels of MYH11, TPM1 and MYL2 were used for electronical verification. In our study, it was found that only MYH11 and TPM1 were expressed in each sample, while the expression level of MYL2 in the sample was 0. Therefore, only statistically significant results were obtained for MYH11 and TPM1. Compared with their expression in the control group, MYH11 and TPM1 were down-regulated in the high-fat and high-energy feed groups, although the results were not significant (Fig. 9). However, the expression trend was consistent with the protein expression results.

### Proteomics verification of MYH11

**Figure 10:**
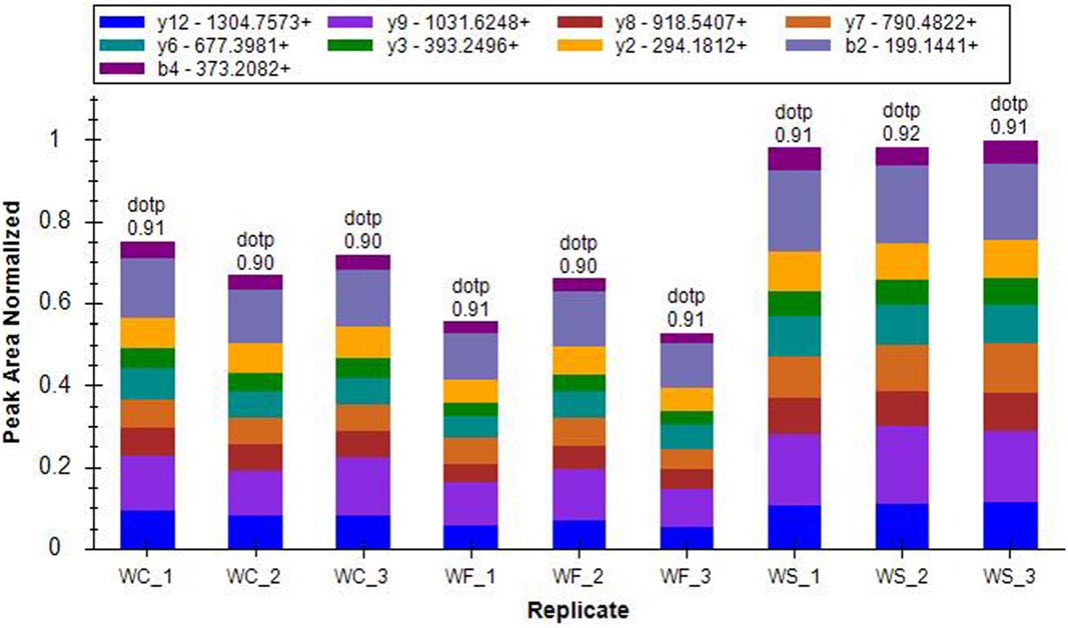
Proteomics verification of the distribution of ion peak area of myosin heavy chain 11 (MYH11) protein in the WC, WF and WS groups. Different colors represented different ion peak areas. In the WF group, the protein levels of MYH11 were significantly decreased. When silybin was added, the protein levels of MYH11 in the WS group increased.

Proteomic verification of MYH11 was performed in the present study. The results showed that the protein expression of MYH11 in the WF group was reduced compared with that of the WC group. The expression of MYH11 in the WS group was notably up-regulated compared with that of the WF group (Fig. 10).

## Discussion

In recent decades, with the increase in the obese population, obesity-related diseases such as type 2 diabetes and cardiovascular diseases have become a considerable threat to people’s health. Obesity-related cardiomyopathy is a typical disease caused by obesity. Previous studies on obesity-related cardiomyopathy mainly focused on cardiac lipotoxicity, metabolic disorders and energy metabolism^5,6^. However, the further research on the specific pathogenesis is still needed. Previous studies indicated that cytoskeletal proteins play a vital role in maintaining myocardial contractile and diastolic functions^13,14^. Using gene chips, Latif et al. found patterns of gene expression changes at the mRNA level of cytoskeletal proteins and non-cytoskeletal proteins in patients who were clinically restored after implantation with left ventricular assist device (LVAD) support. Tropomyosin, myosin light chain and other proteins were up-regulated in patients with clinical implantation of LVAD recovery, suggesting that these proteins play an important role in myocardial reverse remodeling^28^. There are no reports on the effect of silybin on heart-related contractile proteins. In the present study, TMT combined with LC-LC/MS was used to compare the proteomics expression profiles of adipose tissue of healthy, obese and silybin-treated obese mice to identify cardiovascular diseases-related cytoskeletal proteins. It was found that the fat metabolism of mice fed with high-fat diet was abnormal. GO analysis showed that the majority of DEPs were mainly involved in fat metabolism and energy metabolism. Fat metabolism in obese mice was significantly improved after silybin intervention. The above results are consistent with those from our previous study^21^. Thus, a PPI network was constructed. PPI analysis showed that TPM1, MYL2 and MYH11 were highly relevant. These proteins were involved in the differentiation and development of the heart.

TPM is an α-helical coiled protein dimer that binds to actin, forming a continuous polymer along the actin filaments^29^. In mammals, there are four variants of the TPM gene: TPM1/α-TM, TPM2/β-TM, TPM3/γ-TM and TPM4/δ-TM, which generate multiple TPM subtypes by alternative splicing and/or by using different promoters^30^. TPM1 is abundantly expressed in epithelial cells, fibroblasts and smooth muscle cells. In striated muscle, it mainly regulates muscle contraction, while it mainly maintains the cytoskeleton in non-smooth muscle cells. TPM1 is also involved in numerous biological activities in the body, such as cytokinesis, cell membrane material transport, cell movement, maintenance of the cell structural integrity, induction of apoptosis and signal transduction^31^. TPM1 also inhibits cancer cell proliferation and migration, which are associated with the occurrence, development and prognosis of tumors^32–34^. The TPM1 gene encodes fast-twitch skeletal muscle α-TM. Mutations in this gene are associated with hereditary cardiomyopathy by affecting its interaction with actin, as well as Ca^2^ + sensitivity and myofilament contraction rate^35^. Caroline et al. found that Tpm1^-/-^ mice died within 8.5–11.5 days after birth, showing thinner ventricular walls, fewer trabeculae, increased intracellular space, enlarged cell protrusions, and smaller attachments and myofibrils^36^. Missense mutations in TPM1 are associated with hypertrophic cardiomyopathy and dilated cardiomyopathy^37^. The present study found that the expression level of TPM1 was decreased in obese mice fed with a high-fat diet, while it was increased after silybin intervention. Therefore, it was speculated that obesity may damage heart structure and function by down-regulating TPM1 expression, while silybin can protect the heart by up-regulating TPM1 expression. However, the specific mechanism needs further investigation.

MYL2 is a sarcomeric protein with a relative molecular mass of ~ 19,000 Da that belongs to a member of the calcium-binding protein family. In mammals, there are three different gene codes, namely the rapid contraction of skeletal muscle subtype (MLC-2f), the heart ventricle and slow contraction skeletal muscle subtype (MLC-2v) and the cardiac atrial subtype (MLC-2a)^38^. MYL2 encodes myosin regulatory protein (RLC) in the ventricle^39^. RLC is the main regulatory subunit of striated muscle, and it regulates myocardial contraction by regulating troponin/promyosin and Ca^2+^^40^. MYL2 mutations lead to the occurrence of cardiomyopathy^14^. MYL2 is also involved in the occurrence and development of chronic heart failure. In patients with heart failure, the level of MYL2 is reduced, which is associated with the severity of myocardial disease^41^. Previous studies have found that mutations in the MYL2 gene are harmful to HCM^14^. Myosin light chain kinase (MLCK) is the main regulator of the phosphorylation of MYL2. Increased phosphorylation of MYL2 can increase the binding affinity between myosin and actin, thereby accelerating myocardial contraction^42^. Seguchi et al. found that the expression of MLCK and the phosphorylated MYL2 were reduced in the myocardium of patients with heart failure^43^. MYL2 and its phosphorylation levels in the heart of Mlck-deficient mice were decreased, which resulted in cardiac dysfunction, and rapidly progressed to heart failure, with the histological manifestations of myocardial tissue disorder, fibrosis, reduced contractility and cell death. However, over expression of Mlck can attenuate cardiac dysfunction^39,44^. In the present study, the expression of MYL2 was decreased in mice with obesity induced by a high-fat diet. It was suggested that obesity may reduce the binding of myosin and actin, and the regulation of Ca2^+^ by reducing the expression of MYL2, thus causing myocardial contractility disorder, which is involved in the occurrence of obese cardiomyopathy. Significantly, silybin could interfere with this process to protect cardiac function by upregulating the expression of MYL2.

Smooth muscle myosin heavy chain, encoded by MYH11, belongs to the myosin heavy chain family^45^, and functions in contraction, cell migration and adhesion, intracellular transport, and signal transduction^46^. Numerous studies have shown that MYH11 is lowly expressed in tumor tissues, which promotes the proliferation and migration of tumor cells, and is associated with the prognosis of tumors^47^. Vilkas et al. used genomics and proteomics to study the effects of argan extract (AR) on cardiac function caused by pressure overload, and found that ATPase sarcoplasmic/endoplasmic reticulum Ca^2+^ transporting 2 (Serca2), calmodulin 3 (Calm3), MYH11 were up-regulated in the heart tissue of rats that were administered AR, which significantly improved the heart function^48^. Using gene transfection technology, Callie et al. found that over expression of the MYH11 gene at the transcription level could cause endoplasmic reticulum stress, which led to protein degradation by increasing autophagy degradation the level drops abnormally^49^. It was indicated that a decrease in MYH11 protein would lead to endoplasmic reticulum stress and cardiomyocyte damage. On the other hand, atherosclerosis is the pathological basis of coronary heart disease and other types of cardiovascular diseases. Vascular smooth muscle cells (SMC) are key cells in the formation of atherosclerotic plaques. Low expression of MYH11 can promote coronary atherosclerosis and destroy the stability of coronary artery walls, thereby causing coronary artery dysfunction, reducing blood perfusion of myocardial cells, and aggravating the hypoxic damage of myocardial cells. In the present study, the expression of MYH11 in obese mice was decreased. It was suggested that the decrease in MYH11 could affect the heart function by aggravating endoplasmic reticulum stress, promoting cardiomyocyte proliferation and hypertrophy, and affecting myocardial blood flow perfusion. Notably, silybin could up-regulate the expression of MYH11 to interfere with this process to protect heart function.

KEGG analysis of the DEPs was also conducted in the present study. The results showed that the DEPs were enriched in the myocardial contraction, dilated cardiomyopathy and hypertrophic cardiomyopathy pathways (Fig. 5). Importantly, it was found that MYH11, TPM1 and MYL2 were involved in cardiac contraction according to KEGG analysis (Table 1). Calcium regulation of cardiac muscle contraction is controlled by troponin and tropomyosin combined with actin^35^. TPM plays a key role in Ca2 + -mediated regulation of striated muscle contraction by binding to actin and troponin complexes^50^. MYL2 is the main troponin in mammalian striated muscle, which regulates myocardial contraction by regulating troponin/tropomyosin and Ca^2+^^38,40^. MYH11 belongs to the MYH family, which hydrolyzed proteins involved in muscle contraction through adenosine triphosphate^51^.

The present study found that TPM1, MYL2 and MYH11 proteins (involved in myocardial contraction) were down-regulated in mice fed with high-fat diet, while they were up-regulated after silybin intervention. Therefore, it was speculated that TPM1, MYL2 and MYH11 could be the targets of obesity-induced cardiomyopathy, thus providing a theoretical basis for future drug development. However, the present study has certain limitations. Firstly, the number of samples in the electronic verificationis small, causing a certain degree of error. Secondly, the in-depth mechanism of TPM1, MYL2 and MYH11 protein changes in obese cardiomyopathy has not been studied. It was speculated that this may be caused by changes in cardiac metabolism. However, the specific mechanism needs to be further explored.

## Materials and methods

### Laboratory animal

A total of 36 7-week-old clean-grade male C57BL/6 JC mice were purchased from Beijing Viton Lihua Experimental Animal Technology Co., Ltd. and bred in the Animal Experiment Center of the Clinical Research Center of Hebei General Hospital. These mice were housed at a room temperature of 20–25 ˚C, 40–60% relative humidity and 12-h light–dark cycle. After 1 week of adjustable feeding, the animals randomly separated into WC, WF, and WS groups. The animals in the WC group were fed with fodder composed of 70% carbohydrate, 10% fat and 20% protein (gross heating value: 348 kcal/100 g). The WF and WS groups were fed with fodder composed of 20% carbohydrate, 60% fat and 20% protein (gross heating value: 524 kcal/ 100 g). Each group was daily fed with an equivalent calories fodder, and free intake of water. Daily record of food intake was performed. After 4 weeks, mice in the WS group received intragastric administration of 54 mg/kg silybin. In the WC and WF groups, mice were administered intragastric isovolumetric physiology brine. Silybin intervention lasted 4 weeks (Table 3).

**Table 3:** Feeding and treatment strategy of the different groups.

Group	1 week	2–5 weeks	6–9 weeks
WC group	Adaptive feeding	Feeding with fodder composed of 70% carbohydrate, 10% fat and 20% protein	Feeding with fodder composed of 70% carbohydrate, 10% fat and 20% protein + Isovolumetric physiology brine
WF group		Feeding with fodder composed of 20% carbohydrate, 60% fat and 20% protein	Feeding with fodder composed of 20% carbohydrate, 60% fat and 20% protein + Isovolumetric physiology brine
WS group		Feeding with fodder composed of 20% carbohydrate, 60% fat and 20% protein	Feeding with fodder composed of 20% carbohydrate, 60% fat and 20% protein + 54 mg/kg of silybin

All experimental procedures were approved by the Animal Ethics Committee of the Hebei General Hospital (NO.202041), and were carried out in accordance with the Hebei Province Experimental Animal Management Regulations.

### Specimen collection

#### Collection of serum and adipose tissue specimens

The mice were weighed after fasting overnight and abdominally anesthetized with 1% pentobarbital sodium (60 mg/kg). Blood was collected from the retro-orbital sinus and centrifuged at 4 ˚C at 3000 rpm for 20 min for serum collection. The collected serum was stored at −80 ˚C for later use. After blood collection, the epididymal adipose tissue was quickly removed, placed in liquid nitrogen and stored at – 80 ˚C.

### Detection of mouse serum index

Commercially available kits were used for the measurement of blood biochemical indicators^21^. Blood samples were placed in an automatic blood biochemical analyzer (Sysmex Corporation), and the blood biochemical indicators TG, TC, LDL-C and HDL-C were recorded separately.

### Protein purification and sample preparation

A suitable quantity of adipose tissue sample was removed from its – 80 ˚C storage, weighed and ground into a powder. Samples in each group were incubated with 4 volumes lysis buffer (8 mol/l urea, 1% protease inhibitor and 2 mmol/l EDTA), and subjected to ultrasound pyrolysis by centrifugation at 12,000 *g* for 10 min at 4 ˚C. The supernatant was collected, and protein concentration was determined using a BCA kit. The supernatant was added to an ultimate density of 5 mmol/l dithiothreitol, and subjected to reduction at 56 ˚C for 30 min. Subsequently, an ultimate density of 11 mmol/l iodoacetamide was added and incubated at room temperature in the dark for 15 min. Finally, the urea concentration of the sample was diluted to < 2 mmol/l. The sample was incubated with trypsin (trypsin:protein ratio, 1:50) at 37 ˚C overnight. Subsequently, trypsin was added again to the sample (trypsin:protein ratio, 1:100), and the enzymatic hydrolysis continued for 4 h. The peptides digested by trypsin were desalted with Strata X C18 (Phenomenex) and freeze-dried under vacuum. The peptides were dissolved with 0.5 mmol/l tetraethylammonium bromide. The labeling reagent was dissolved with acetonitrile, mixed with the peptides and incubated at room temperature for 2 h. The labeled peptides was desalted and freeze-dried under vacuum.

### LC–MS/MS analysis

The peptides were dissolved in liquid chromatography (LC) mobile phase A [aqueous solution containing 0.1% formic acid solution and 2% acetonitrile (v/v)] and separated using the EASY-nLC 1200 ultra-high-performance LC system. Mobile phase B was an aqueous solution containing 0.1% formic acid and 90% acetonitrile. After separation by the aforementioned ultra-high performance LC system, the peptides were injected into the nanospray ionization (NSI) ion source for ionization and then analyzed by Q EXACTIVE HF-X MS. Quantitative experiments for mass detection of total protein were repeated three times.

### Differential protein screening and bioinformatics analysis

MS data were retrieved using MaxQuant (http://www.maxquant.org/). All results of MS detection were simultaneously evaluated by the reverse database search method to evaluate the false positive rate (FPR) of data due to random matching. The FPR of proteins and peptides was < 1%. Under the condition of FPR < 1%, a protein with ≥ 2 identified unique peptides was considered reliable. DEPs were identified and compared in the WC, WF and WS groups. UniProt-Gene Ontology (GO) Annotation (www.http://www.ebi.ac.uk/GOA/) database and InterProScan (https://www.so.com/link) software were used for GO enrichment, KEGG pathway (http://www.kegg.jp/kegg/mapper.html) and cluster analyses. Fisher’s exact test was used to evaluate DEPs. P < 0.05 was considered to indicate a statistically significant difference.

### PPI network analysis

The PPI network was constructed using STRING software (v.10.5, http://string-db.org/). Interaction between proteins was obtained according to a confidence score > 0.7 (high confidence).

### In vitro validation of the mRNA expression of DEPs

Total RNA was extracted using TRIZOL (Qiagen GmbH). Sensiscript RT Kit (Thermo Fisher Scientific. Inc.) was used to synthesize cDNA. Subsequently, RT-qPCR was performed using SuperReal PreMix Plus (SYBR Green) (FP205; Tiangen Biotech Co., Ltd.). β-actin was used as an internal reference for gene detection. The relative gene expression levels were calculated using the 2^-ΔΔCt^ method^52^.

### Verification of mRNA expression of DEPs in the GSE120748 dataset

The GSE120748 dataset was retrieved from the Gene Expression Omnibus (GEO) database^53^. Mice were fed with different feeding regimes for 180 days. The group fed with standard chow was used as the control group, while the high-fat and high-energy chow groups were used as the experimental groups. The number of blood samples in each group was 4.

### Proteomics verification

In this study, parallel reaction monitoring (PRM) was used to verify the identified DEPs. The peptides were dissolved in LC mobile phase A and separated using the EASY-nLC 1000 ultra-high performance LC system. The liquid gradient setting of B was as follows: 6–25% for 0–40 min; 25–35% for 40–52 min; 35–80% for 52–56 min; and 80% for 56–60 min. The flow rate was maintained at 400 nl/min. After separation by the aforementioned ultra-high performance LC system, the peptides were injected into the NSI ion source for ionization and then analyzed by Q EXACTIVE PLUS MS.

### Statistical analysis

Statistical analysis was performed with SPSS.20 software (IBM Corp.). The data are presented in quartile intervals. Analysis of variance was used for comparison between groups when the data satisfied a normal distribution and the variance was uniform. In addition, the non-parametric Student’s t-test was used. P < 0.05 was considered to indicate a statistically significant difference.

### Ethics approval and consent to participate

All experimental procedures were approved by the Animal Ethics Committee of the Hebei General Hospital (NO.202041), and carried out accordance with the Hebei Province Experimental Animal Management Regulations.
